# Development of MijnAVL, an Interactive Portal to Empower Breast and Lung Cancer Survivors: An Iterative, Multi-Stakeholder Approach

**DOI:** 10.2196/resprot.3796

**Published:** 2015-01-22

**Authors:** Wilma Kuijpers, Wim G Groen, Hester SA Oldenburg, Michel WJM Wouters, Neil K Aaronson, Wim H van Harten

**Affiliations:** ^1^The Netherlands Cancer InstituteDivision of Psychosocial Research and EpidemiologyAmsterdamNetherlands; ^2^The Netherlands Cancer InstituteDivision of Surgical OncologyAmsterdamNetherlands; ^3^University of TwenteDepartment of Health Technology and Services ResearchEnschedeNetherlands

**Keywords:** cancer survivors, interactive portal, development, usability testing, empowerment

## Abstract

**Background:**

MijnAVL (MyAVL) is an interactive portal being developed to empower cancer survivors. Literature review and focus groups yielded the selection of features such as access to the electronic medical record (EMR), patient reported outcomes (PROs) and related feedback, and a physical activity support program.

**Objective:**

Our aim was to present a final design of MijnAVL based on (1) health professionals' evaluation of proposed features, (2) cancer survivors’ evaluation of a first draft, and (3) cancer survivors’ evaluation of a functional online prototype.

**Methods:**

Professionals from various disciplines gave input to the content of and procedures related to MijnAVL. Subsequently, 16 cancer survivors participated in an interview to evaluate content and graphic design of a first draft (shown with screenshots). Finally, 7 survivors participated in a usability test with a fully functional prototype. They performed predefined tasks (eg, logging in, finding a test result, completing a questionnaire) while thinking aloud. Descriptive statistics and simple content analysis were used to analyze the data of both the interviews and the usability tests.

**Results:**

Professionals supported access to the EMR (eg, histology reports, lab results, and their letters to general practitioners). They also informed the development of PROs and the physical activity support program. Based on the first draft, survivors selected the preferred graphic design, approved the features and provided suggestions for the content (eg, explanation of medical jargon, more concise texts, notification by emails). Usability tests revealed that it was relatively easy to navigate the website and use the different features. Recommendations included, among others, a frequently asked questions section and the use of hyperlinks between different parts of the website.

**Conclusions:**

The development of MijnAVL, an interactive portal to empower breast and lung cancer survivors, was performed iteratively and involved multiple groups of end-users. This approach resulted in a usable and understandable final version. Its effectiveness should be determined in further research.

## Introduction

### Background

People diagnosed with cancer or who have been successfully treated for cancer (cancer survivors) often experience a range of physical (eg, fatigue, pain) and psychosocial (eg, distress, disruption of work and social relationships) health problems as a result of their disease and its treatment [1]. The majority of cancer survivors want to know as much as possible about their diagnosis, treatment, side effects, and health promotion [2,3]. Also, although we know that physical activity can have positive effects on both the physical and psychosocial well-being of cancer survivors [4,5], many survivors do not meet physical activity norms [6,7]. To cope with the cancer-related problems, fulfil information needs, and promote physical activity, efforts are required to enhance patients’ knowledge, skills, and motivation to positively influence their health, often referred to as patient empowerment [8].

In the Netherlands, 94% of the population has daily access to the Internet. Among the elderly, this varies from 20% (age 75 and older) to 55% (age 65-75), and it is expected that these figures will continue to increase [9]. Apart from information, online interventions can be provided via the Internet, and these eHealth initiatives are likely to increase patient empowerment [10]. To empower breast and lung cancer survivors of the Antoni van Leeuwenhoek hospital, part of the Netherlands Cancer Institute (a comprehensive cancer center in Amsterdam), we are developing “MijnAVL” (MyAVL), a personal, interactive portal. In developing the portal, we have initially focused on the population of breast and lung cancer survivors because of their distinct disease characteristics, impairments, rehabilitation needs and options, and the relatively high incidence of these tumors [11]. The focus on breast and lung cancer relates primarily to the specific content of the portal, but not to its design features or overall functionality. We expect that the design and functionality of the portal will be applicable to a wide range of cancer survivors.

### Features of MijnAVL

The features of MijnAVL are primarily based on a literature review of Web-based interventions for patient empowerment and physical activity in various chronic diseases. We identified features of interactive portals that are likely to be relevant for cancer survivors, including education, self-monitoring, feedback (tailored information), self-management, personal exercise program, and communication with professionals and/or fellow patients [12]. Subsequent focus groups revealed that health professionals were most interested in portal features that would provide them with relevant information about the health status of their patients, while cancer survivors preferred the features that could fulfil their information needs. These preferences led to the following set of requirements for MijnAVL: (1) patient education: relevant information about diagnostic tests, treatments, and rehabilitation opportunities, (2) an overview of past and future appointments, (3) access to the electronic medical record (EMR): reports of diagnostic tests and lab tests, letters to general practitioners, etc, (4) patient-reported outcomes (PROs) and related feedback for patients: symptom and health-related quality of life outcomes including a summary of scores, accompanied by relevant information on the different outcomes (eg, background information and tips for fatigue), and (5) a physical activity support program: tailored physical activity advice based on a set of questionnaires.

Survivors can access MijnAVL by providing their DigiD username and password accompanied by a code that is sent to their mobile phone. DigiD is a safe authentication method related to one’s social security number, that is used primarily (but not exclusively) by various governmental services.

The objective of the current study was to describe the development of MijnAVL by “translating” the proposed set of requirements into final content and design based on (1) health professionals’ evaluation of content, (2) cancer survivors’ evaluation of a first draft of screenshots (interviews), and (3) cancer survivors’ evaluation of a functional prototype through usability tests.

## Methods

### Overview

MijnAVL was developed using a stepwise approach involving both health professionals and cancer survivors from our institute, as this increases the likelihood of the portal being accepted and actually used in daily practice [13,14]. The Institutional Review Board exempted this study from formal review, and all cancer survivors provided written informed consent.

### Health Professionals’ Evaluation of Content

We obtained health professionals’ feedback during various sessions in which the proposed portal features were presented in PowerPoint. With regard to access to the EMR, we organized group sessions for breast and lung cancer separately, and in both sessions approximately 10 professionals participated (medical oncologists, surgeons, radiotherapists, nurse practitioners). We discussed which parts of the EMR should be accessible and under what conditions. After we had constructed a first draft of MijnAVL, these professionals participated again in a group session. Seven professionals joined a group session to discuss when PROs should be completed and what type of feedback should be provided to both health professionals and survivors. In addition, we had individual interviews with a medical oncologist, 2 surgeons, a nurse practitioner, and a social worker about the feedback that could be provided based on PRO scores. The advice to be given in the physical activity support program was written and discussed in collaboration with 5 physical therapists and a rehabilitation physician.

### Cancer Survivors’ Evaluation of a First Draft (Semistructured Interviews)

Cancer survivors were eligible when they were adult, under curative treatment or within 3 years of treatment completion, had a basic fluency in Dutch, and had at least minimal experience with computers and Internet. We organized semistructured interviews until saturation of qualitative data occurred (ie, no new information came up) [15]. Ten breast and 6 lung cancer survivors with a mean age of 61.4 (SD 9.8, range 45-77) years participated; 75% of participants were female. The majority of the survivors were highly educated, and 14 individuals had been using the Internet daily for more than 2 years.

Participants completed a questionnaire on sociodemographics and verbally consented to audiotaping of the session. Subsequently, the researcher (WK or WG) presented screenshots with two types of graphic design and examples of the intended content of MijnAVL. In a semistructured interview, we focused on portal design and content, especially regarding access to the EMR, PROs, and the physical activity support program. Participants also reported what they considered to be the most positive and negative aspects of the portal and suggestions for possible improvements.

The interviews were analyzed by a simple content analysis [16] of the notes taken by the researcher and, if notes were not sufficient, listening back to the audiotapes. The data were structured according to each feature of MijnAVL or the category graphic design. The results from these semistructured interviews were used to develop a functional prototype of the portal. Before conducting the usability tests, the technical functioning of MijnAVL was rigorously tested in numerous iterations by the research team.

### Cancer Survivors’ Evaluation of a Functional Online Prototype (Usability Tests)

Eligibility criteria and recruitment procedures were identical to those of the semistructured interviews. We organized individual sessions in a lab setting until saturation occurred [15]. Five female breast cancer and 2 male lung cancer survivors, with a mean age of 50.6 (SD 5.7, range 44-61) years, participated in a usability test. They all had been using the Internet for more than 2 years, and about half had graduated from college or university.

First, participants completed a questionnaire on sociodemographics and agreed to audiotaping of the session. Then they performed various tasks, including logging on to the portal, completing a questionnaire digitally, checking future appointments, and finding the results of a lab test. During task performance, they were encouraged to think aloud, which provided insight into how they were performing these tasks [17]. After completion of the tasks, the researcher asked about the positive and negative aspects of the portal. Finally, participants completed a questionnaire on their expectations regarding the portal, based on the Unified Theory of Acceptance and Use of Technology (UTAUT) [18], and on anticipated effects on patient empowerment, based on the Patient Activation Measure [19]. Data analysis was similar to that of the interviews, except for adding usability as a category to organize the data. Results were used to produce the final design of MijnAVL.

## Results

### Health Professionals’ Evaluation of Content

#### Access to the Electronic Medical Record

The majority of health professionals supported patients’ access to the reports of lab tests, histological examinations, and letters from the hospital to general practitioners. However, they expected that this information would evoke anxiety and many questions. Therefore, professionals involved in lung cancer care especially had reservations about showing radiology reports, specifically due to ambiguous test results and the generally poor prognosis of lung cancer survivors. They indicated several conditions for providing test results: development of an accompanying medical dictionary, introductory texts for each report, and a delay of 2 weeks before sharing the test results online (to ensure that test results are personally discussed with patients first).

#### Patient Reported Outcomes and Related Feedback

Health professionals recognized the potential benefits of PROs (being aware of a patients’ symptoms and quality of life), but they also expected an increased workload due to having to discuss these various issues with their patients in more detail than might typically be the case. Patients should preferably complete PROs at diagnosis, after completion of treatment, and at each follow-up visit. Health professionals preferred having PRO data summarized graphically, with clear indication of problem areas (especially worsening of symptoms), the clinical significance of scores, and suggestions for referral. They were reluctant about automated advice to patients about different symptoms because they believed that the etiology of many symptoms is too complex to allow for standardized advice. It was therefore decided to provide patients with an overview of the PRO results, supplemented by more general information and advice related to each domain of the questionnaire. For example, if survivors reported clinically relevant levels of fatigue, they would be directed to information on cancer-related fatigue. Professionals from all relevant disciplines assisted in drafting this type of information.

#### Physical Activity Support Program

Because the support to be offered in the program is relatively non-specific (ie, it is aimed at increasing general levels of physical activity), we opted for a stand-alone, computerized system (providing advice based on a questionnaire). It was decided to cover the following factors in the physical activity questionnaire: “stage of change”, nutritional status, possible contraindications for physical activity, treatment phase (during or after treatment), tumor type (breast or lung cancer), whether the patient is participating in a supervised exercise program, and if yes, whether additional information on physical activity is desired. Two well-known theoretical models were used to generate the physical activity advice: Social Cognitive Theory [20] and Theory of Planned Behavior [21]. All information and advice was written in a collaboration between the researchers, the physical therapists, and the patient education service of the hospital.

### Cancer Survivors’ Evaluation of a First Draft (Semistructured Interviews)

#### Overview

In general, participants were positive about MijnAVL and the majority of them were familiar with the DigiD authentication method to log in. Major positive aspects of MijnAVL were involvement in the health care process and the accessibility of information (transparency), whereas negative aspects were too lengthy texts and use of medical jargon.

#### Access to the Electronic Medical Record

Access to the EMR was appreciated, but concerns were raised about being able to understand the information provided and the potential worry or upset that could result. These concerns appeared to be justified, as most patients understood less than half of the information provided in the radiology and pathology reports. However, this improved when they were provided with a dictionary of terms. Results of clinical lab tests were easier to understand because reference values were already provided in the tables. Some participants wanted to receive additional contextual information*,* for example, which specific blood test values indicate that one is able to receive a new cycle of chemotherapy or how blood markers are related to lifestyle factors (such as cholesterol levels). Access to the letters to the general practitioner were considered as a good summary by some, and one participant wanted to be provided with the treatment plan and results from multidisciplinary meetings.

#### Patient Reported Outcomes and Related Feedback

Participants wished to receive a notification (eg, text message, email, or a notification on the homepage of MijnAVL) when they needed to complete a questionnaire. Most participants were able to understand graphical summaries of the PROs, and there was no clear preference for line charts versus bar charts. The majority wanted to see changes over time, both positive and negative.

#### Physical Activity Support Program

Many of the participants (11/16) expected that this would increase awareness of the importance of being physically active during and directly after treatment. Five were already quite physically active and would not need such a program. Participants expected to receive information on which activities are allowed (or discouraged) during treatment, and more personal information on rehabilitation and reimbursement of rehabilitation programs. The examples of this type of information and advice were judged to be interesting, understandable, reliable, of appropriate length, and quite motivating. An example of a graph presenting the amount of physical activity undertaken (based on self-report) during the past week was relatively easily understood.

#### Graphic Design

The preferred homepage contained large buttons with icons that indicated the features of MijnAVL; this was perceived as highly accessible. The favored content page was divided into several blocks of main text with the possibility to click for more information. Participants liked that the information was illustrated by visual images. Recommendations and resulting changes in portal design and content are shown in Table 1.

**Table 1:** Recommendations and changes in portal design and content based on semistructured interviews with cancer survivors (N=16).

Recommendation		Adjustment(s)
**Graphic design**		
	The term “physical activity advice” is not very appealing.	Physical activity advice was changed to “keep fit”.
	The overall look of MijnAVL is somewhat boring.	A photo was added to the background to improve the overall visual attractiveness.
	The large red text box indicating patient number, name, and date of birth should be displayed on the homepage only.	The text box was deleted from all pages except for the homepage. Instead, identical information was displayed on top of the menu on the left.
	Provide the specific location in the hospital in the overview of appointments.	None (technically not possible).
**Content: Access to the EMR**		
	Provide detailed information about the different elements of the blood (What does that abbreviation mean? What is the function of this element?).	A link to the website of the Dutch Society of Clinical Chemistry and Laboratory Medicine (NVKC), containing this information, was included.
	The information from the EMR should be provided in a way that a layman can understand.	A medical dictionary was created.
**Content: PROs and related feedback**		
	It is necessary to receive a notification when you have to fill out a questionnaire.	An email will be sent when a new questionnaire needs to be filled out.
**Content: Physical activity support program**		
	The explanatory text accompanying the graph with physical activity information is too complicated.	It is technically not (yet) possible to include graphs, so the information is provided in a table. The explanatory text was adjusted and simplified accordingly.

### Cancer Survivors’ Evaluation of a Functional Online Prototype (Usability Tests)

#### Overview

More than half of participants (5/7) mentioned the overview of appointments to be the most positive aspect of MijnAVL, followed by access to the EMR, the reliability of the information, and the fact that all information is gathered at a single location. Less positive aspects included concerns about possible computer hacking and the comprehensibility of information from the medical record. MijnAVL was rated as 7.9 on a 10-point scale (10=excellent). Suggestions for additional features included an option to ask questions online (e-consult) and a frequently asked questions section (with both portal-related and cancer-related issues).

#### Access to the Electronic Medical Record

Participants highly appreciated the accessibility of information from the EMR, especially the possibility of reading over information. The standard dictionary with medical terms on MijnAVL was helpful but did not contain all words that participants were looking for. The clinical lab results were relatively easy to understand, although deviating values should be highlighted.

#### Patient Reported Outcomes and Related Feedback

Participants were content with the possibility of completing PROs online instead of in the hospital. There were some complaints about response options, and 3 participants thought that the questionnaire was too long. However, as we are using validated questionnaires, these factors could not be changed. All were able to interpret their scores per domain as shown in the tables and valued the availability of information and advice on many aspects of quality of life.

#### Physical Activity Support Program

Five participants thought that they would benefit from the tailored advice of the program; the remaining two indicated that they were already intrinsically motivated to be physically active. One explicitly mentioned that it is good to incorporate a specific goal such as the Dutch Norm of Physical Activity. Some questions of the questionnaire related to the program were hard to answer, for example, on the amount of physical activity in the past week (recall problems). The table showing the amount of high and moderate intensive exercise was clear to everyone.

#### Graphic Design

Participants were satisfied with the clear, simple, and accessible layout. Letter type and font size were good, and the menu bar on the top of the page was perceived as convenient. It was recommended to place the most important information at the top of each page and keep the information as concise as possible. Many participants noted that they were inclined to skip the introductory texts and first look for the “real” information (eg, a table with their personal quality of life scores).

#### Usability

All participants managed to log in to the website, although one experienced problems due to the browser used. They were able to navigate through MijnAVL with little or no help and typically used the menu bar on top of the page to go to other features of MijnAVL. The menu on the left was used to navigate within a single feature. Participants made several navigation errors. One tried to find the results from a mammogram by clicking on the appointment during which the mammogram was taken, while this information could be found in the EMR. Three experienced difficulties with finding the quality of life questionnaire to be filled out, as well as with finding the additional information on quality of life domains. Most of them first visited the “Keep fit” page to find the physical activity questionnaire, as they associated this with physical activity. However, this one could actually be found under the button “questionnaires”. The final task, logging out from MijnAVL, was not a problem. An overview of recommendations and adjustments based on the functional prototype are shown in Table 2.

#### Expectations

Table 3 shows the results of the questionnaire on expectations of MijnAVL and expected effects on patient empowerment.

**Table 2:** Recommendations and adjustments based on usability tests (N=7).

Recommendation		Adjustment(s)
**Graphic design**		
	The button to log in should be placed on top of the login page.	The button to log in was moved to the top of the login page, followed by instructions on what to do when you forgot your password or need to request a DigiD account.
	Introductory and/or explanatory information should be provided in a pop-up.	None (technically not possible yet).
**Content: Access to the EMR**		
	The denomination “medical imaging” is not clear and should be changed to “radiology”.	This term was not changed, as “medical imaging” also contains the results of nuclear medicine.
	The meaning of the letters L (low) and H (high) accompanying clinical lab results should be elucidated.	The introductory text was extended with a sentence explaining the meaning of L and H.
	It would be convenient if you could search in the medical dictionary.	Planned for a next phase (technically not possible yet).
	The menu on the left should contain only the test results that you have actually received.	Planned for a next phase (technically not possible yet).
**Content: PROs and related feedback**		
	You have to scroll a lot when filling out the questionnaires.	None (technically not possible).
	The educational materials should be placed in alphabetic order.	Planned for a next phase (technically not possible yet).
**Content: Physical activity support program**		
	A hyperlink to the physical activity questionnaire is required on the “Keep fit” page when physical activity support has not yet been provided.	This hyperlink was added.
**User performance**		
	More direct hyperlinks between the different features would be helpful.	These hyperlinks were added where possible.
	A section with frequently asked questions would be helpful.	A list with frequently asked questions related to login procedures and who to approach in case of questions was added.
	Informational movies would make MijnAVL more attractive.	Planned for a next phase (due to time restrictions).

**Table 3:** Expectations based on UTAUT and expected effects on patient empowerment (N=7).

		Negative^b^, n	Neutral^b^, n	Positive^b^, n
**Statements based on UTAUT** ^a^				
	Using MijnAVL will contribute positively to my feelings of control with regard to my health status	1	1	5
	MijnAVL will be easy to use	0	0	7
	People who are important to me would think that I should use MijnAVL	3	4	0
	I have the knowledge and resources to use MijnAVL	0	0	7
	I will be able to use MijnAVL	0	0	7
	I feel positive about MijnAVL	0	0	7
	Using MijnAVL will evoke emotional feelings	2	1	4
	I intend to use MijnAVL	0	1	6
**Statements related to patient empowerment** ^a^ **: “By using MijnAVL…”**				
	…my knowledge about my disease, treatment, and effects will increase	0	3	4
	…I can take an active role in my own health care	1	4	2
	…I am confident I can help to prevent or reduce problems associated with my health	2	3	2
	…I am confident that I can tell whether I need to go to the doctor or whether I can take care of a health problem myself	4	0	3
	…I am confident that I can tell a doctor concerns I have even when he or she does not ask	1	2	4
	…I will be motivated to maintain lifestyle changes	1	6	0

^a^All statements were rated on a 5-point scale ranging from 1 (completely disagree) to 5 (completely agree).

^b^Negative=score 1 + 2; Neutral=score 3; Positive=score 4 + 5.

## Discussion

### Principal Findings

**Figure 1:**
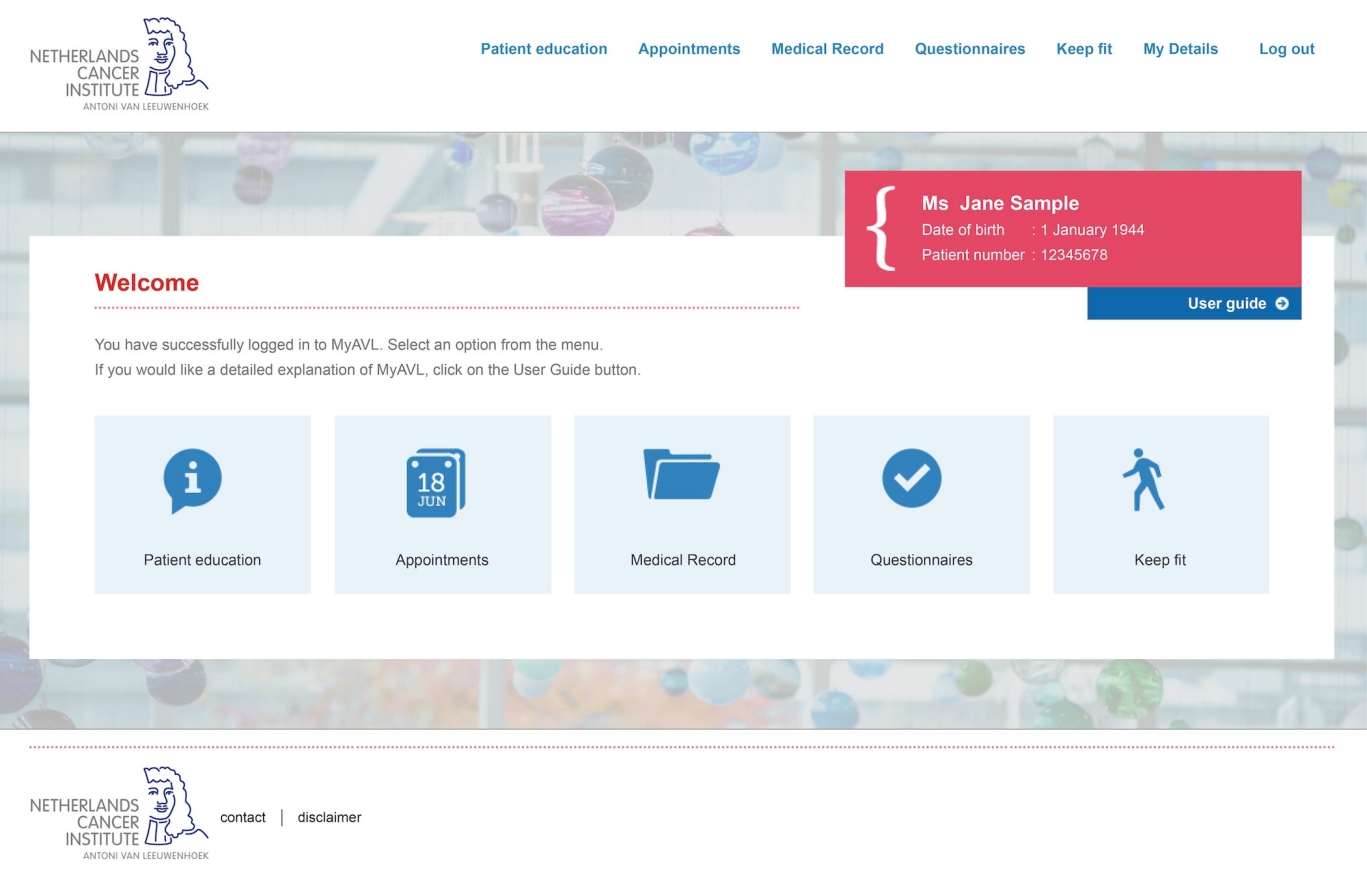
MijnAVL: Homepage.

**Figure 2:**
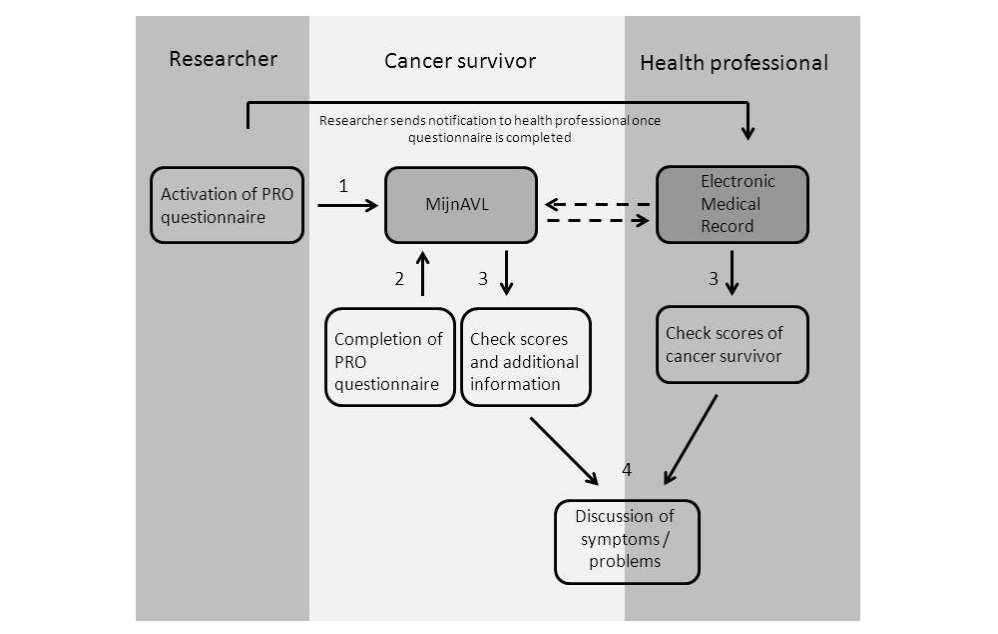
Interactive feature of MijnAVL: PROs.

**Figure 3:**
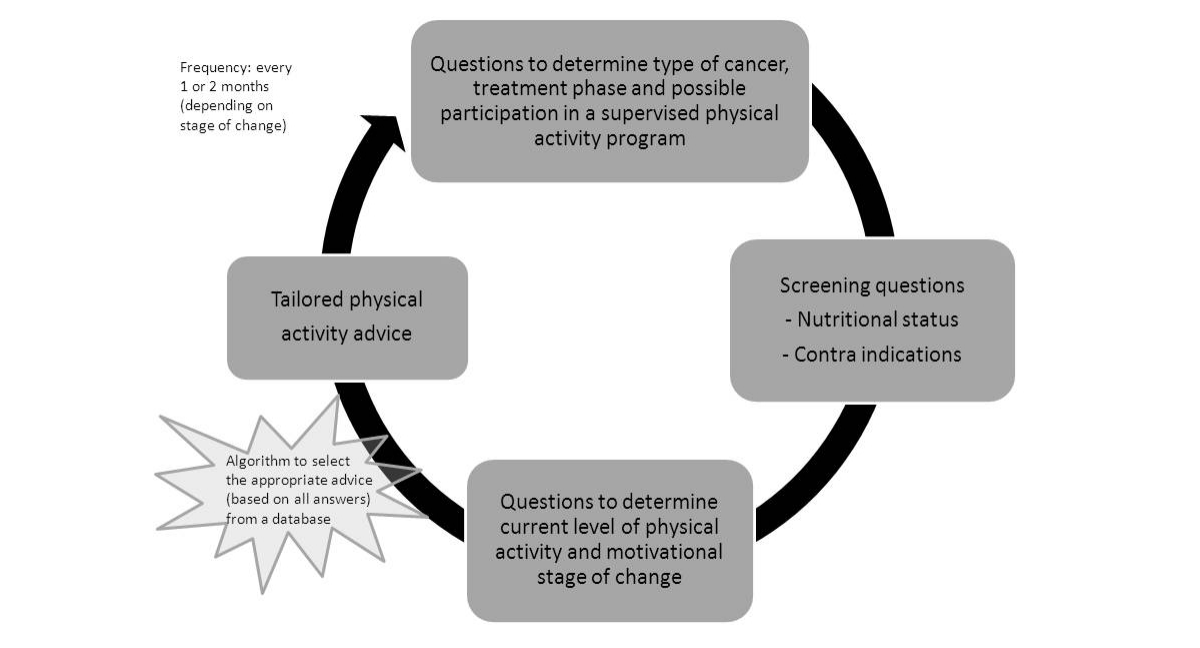
Interactive feature of MijnAVL: physical activity support program.

In this paper, we have described the development of the final design of MijnAVL, an interactive portal to empower cancer survivors. Health professionals provided feedback on the proposed content of and conditions required for MijnAVL, and contributed to the development of information and advice. Cancer survivors participated in interviews to evaluate a first draft of screenshots and performed usability tests with a functional prototype. Their suggestions concerning graphic design, content, and usability were taken into account as much as possible in generating the current version of MijnAVL, within the limits imposed by technology and time (see Figures 1-3 and Multimedia Appendix 1).

In general, both health professionals and cancer survivors were positive about the development of MijnAVL. Cancer survivors appreciated the features that provided them with relevant information and especially the overview of appointments and access to their EMR. However, it is known that patients, in general, do not fully understand their medical records [22]. This could explain why they indicated that a dictionary or some other type of aid was needed to understand all the information in the EMR. Other studies have also shown that an explanation of medical jargon, abbreviations and acronyms, and additional information on tests and results [23] or an online dictionary [24] are useful and necessary tools. Although the participants in this study have provided a suggestion in this regard (eg, that it should be possible to search in the dictionary), further research is needed to determine the specific content and optimal format of these tools.

Related to this, health professionals expected that patients’ access to the EMR would lead to an increased workload, because patients would find it difficult to understand the medical information and it could evoke anxiety and many questions. Indeed, based on a usability test, study participants expected that using MijnAVL would evoke emotional feelings. However, these expectations may not be justified, as several studies have shown that giving cancer patients access to their EMR does not increase anxiety levels [25,26]. Furthermore, Rodriguez et al [27] have shown that, according to 75% of the physicians and nurses participating in their study, workload did not increase after the implementation of patients’ access to laboratory results. The fear of an increased workload also applied to the introduction of PROs, due to the expected time needed to prepare consultations and discuss results. However, previous trials have shown that the use of PROs does not increase the duration of consultations [28,29].

Survivors’ feedback regarding PROs and the physical activity support program was more diverse. While the majority saw value in these features of the portal, some did not. It is important that participants not only value the usability of MijnAVL, but that they are also aware of its aim (increasing patient empowerment). In this study, survivors anticipated that using MijnAVL would only moderately improve empowerment (Table 3). It may have been difficult for them to judge the actual value of MijnAVL and its interactive features based on a usability test only. An alternative explanation could be that the features need further adjustment and fine-tuning to enhance their effectiveness.

Our results indicated that professionals were primarily interested in PRO scores indicating a worsening of symptoms, whereas survivors preferred to see both worsened and improved scores. This could be due to their different perspectives: professionals need to help the patient to reduce symptom burden, while survivors apparently want to monitor changes in their symptom experience and functional health, both positive and negative. When feeding back the results of PROs to health professionals and survivors, it is important to take into account their unique preferences. What also needs to be fine-tuned, is the way that information is provided. For example, survivors suggested that educational materials should be placed in alphabetic order, which is not yet possible due to technical restrictions. However, we acknowledge the importance of providing information in the most convenient manner.

### Limitations

Several possible limitations of this study need to be considered. First, the number of participants in this study was relatively small (16 and 7 participants in the interviews and usability tests, respectively). However, it is known that serious problems are most likely to be discovered with the first participants, and the last sessions did not reveal any new issues (saturation), so we assume that these numbers were sufficient [30]. Second, participants were probably more likely to be interested in and able to use MijnAVL than those who declined (response bias). Indeed, important reasons for non-participation were not having a computer and disinterest, suggesting that our sample may well be representative of the target population of computer literate patients who have interest in such a system. It is a challenge to motivate and enable the non-participants to use MijnAVL in order to benefit from its features. Finally, this was a single center study conducted in a specialized cancer center. The results may not be entirely applicable to other settings, in which the treatment of cancer is just one of many different disciplines.

### Strengths

An evident strength was that we used a multi-stakeholder approach in which all relevant end-users were involved. Both health professionals and cancer survivors provided feedback during the developmental process of MijnAVL, ensuring that the portal fits their needs as much as possible. Second, the development of MijnAVL included multiple iterations, with the results of the previous iteration being incorporated in the next prototype. We were able to detect possible problems regarding content and design at an early stage and to adjust MijnAVL accordingly, resulting in a user-friendly portal.

Although similar initiatives have been undertaken successfully in other chronic disease areas such as diabetes, congestive heart failure and chronic obstructive pulmonary disease [12,31,32], the use of information technology to enhance patient empowerment is relatively new in the field of oncology. The features of MijnAVL reflect different trends that are currently seen in oncology, such as electronic symptom monitoring (eg, [33,34]), hospital-based systems to provide patient education, and to a lesser extent, access to medical record information. MijnAVL incorporates these various features, targeting different aspects of cancer survivorship simultaneously. We anticipate that the portal will enhance empowerment by increasing survivors’ knowledge and understanding of their disease and its treatment, by providing personally relevant information, by allowing them to self-monitor their symptoms and functional health, and by providing tools for becoming more physically active. We are currently conducting a pilot study to evaluate the effects of MijnAVL on empowerment and satisfaction.

### Conclusions

In this paper, we have described an iterative approach, involving all relevant stakeholders and end-users, to develop MijnAVL. Content and usability were improved based on the feedback of participants, which has resulted in a user-friendly portal with the potential to empower cancer survivors. Ongoing and future research is needed to evaluate the efficacy of MijnAVL in daily clinical practice in terms of meeting the information needs of cancer survivors and health professionals, in enhancing patient empowerment, and ultimately in contributing to the quality of life of cancer survivors.

## Multimedia Appendix 1

Screenshots of MijnAVL.

## Abbreviations

EMR: electronic medical record
PROs: patient-reported outcomes
UTAUT: Unified Theory of Acceptance and Use of Technology
