# Supplementary material for: Development of MijnAVL, an Interactive Portal to Empower Breast and Lung Cancer Survivors: An Iterative, Multi-Stakeholder Approach
Source: JMIR Res Protoc. 2015 Jan 22;4(1):e14. doi: 10.2196/resprot.3796 (PMC4319089; doi:10.2196/resprot.3796)
Supplement: Supplementary file 1 [file resprot_v4i1e14_app1.pdf]

Ms Jane Sample
Date of birth : 1 January 1944
Patient number : 12345678

User guide

Welcome

You have successfully logged in to MyAVL. Select an option from the menu.
If you would like a detailed explanation of MyAVL, click on the User Guide button.

Patient education

Appointments

Medical Record

Questionnaires

Keep fit

Ms Jane Sample
Patient number : 12345678

Patient education

- New information
- My information
- - General
- - Diagnostics
- - Diagnosis
- - Treatment / treatment plan
- - Complaints and symptoms
- - Rehabilitation, counselling and support
- - Survivorship care
- - Study
- - Hereditary
- My important information
- Find information

Complaints and Symptoms

Print

All the information, categorised as complaints and symptoms, can be found under 'Complaints and Symptoms' in your personal library. You can add search words to your information to help you retrieve these quickly using the search options.

Mark information as 'important' to help you retrieve it easily under 'My important information'.

☑ Also show items read

Subsequent check and follow-up on lung cancer !
Assigned by Average, Joe on 9 April 2014
Tags: Edit

Mark as unread
Delete marked as 'important'

Painful mouth
Assigned by Average, Joe on 9 April 2014
Tags: Edit

Mark as read
Mark as important

Painful eyes
Assigned by Average, Joe on 9 April 2014
Tags: Edit

Tingling hands and feet
Assigned by Average, Joe on 9 April 2014
Tags: Edit

Fatigue
Assigned by Average, Joe on 9 April 2014
Tags: Edit

Constipation
Assigned by Average, Joe on 9 April 2014
Tags: Edit

Pain

Ms Jane Sample
Patient number : 12345678

Appointments

- Future appointments
- Past appointments

Future Appointments

Print

All your scheduled appointments can be viewed here. If you wish to view your past appointments, click on Past Appointments.

Day	Date	Time	Virtual Time		
Monday	23-06-2014	08:00	08:00	Radiology (CareStream)	MRI06
Monday	23-06-2014	08:00	08:00	Radiology (CareStream)	MRI11
Monday	23-06-2014	07:00	07:00	Radiology (CareStream)	Mamm01
Donderdag	26-06-2014	07:00	07:00	Radiology (CareStream)	Mamm01
Thursday	30-06-2014	07:00	07:00	Radiology (CareStream)	MRI06
Monday	30-06-2014	07:00	07:00	Radiology (CareStream)	MRI11
Monday	30-06-2014	07:00	07:00	Radiology (CareStream)	Mamm01
Thursday	03-07-2014	07:00	07:00	Radiology (CareStream)	Mamm01
Monday	07-07-2014	07:00	07:00	Radiology (CareStream)	Mamm01
Thursday	10-07-2014	07:00	07:00	Radiology (CareStream)	Mamm01
Monday	14-07-2014	07:00	07:00	Radiology (CareStream)	Mamm01
Thursday	17-07-2014	07:00	07:00	Radiology (CareStream)	Mamm01
Monday	21-07-2014	07:00	07:00	Radiology (CareStream)	Mamm01
Thursday	24-07-2014	07:00	07:00	Radiology (CareStream)	Mamm01
Monday	28-07-2014	07:00	07:00	Radiology (CareStream)	Mamm01
Thursday	31-07-2014	07:00	07:00	Radiology (CareStream)	Mamm01
Monday	04-08-2014	07:00	07:00	Radiology (CareStream)	Mamm01
Thursday	07-08-2014	07:00	07:00	Radiology (CareStream)	Mamm01
Monday	11-08-2014	07:00	07:00	Radiology (CareStream)	Mamm01
Thursday	14-08-2014	07:00	07:00	Radiology (CareStream)	Mamm01

First | ~~Previous~~ | Next | Last

Ms Jane Sample
Patient number : 12345678

Medical Record

- Pathology results
- Imaging tests
- Lab results
- ECG (electrocardiogram/heart test)
- Ergometry (bicycle test)
- Lung function test
- Quality of life scores
- Operations
- Letters
- Medical glossary

Medical Record

Print

The results of tests you have undergone can be found under Medical Record. Select an option from the various test results and reports from the vertical menu on the left.

The components shown in your medical record must be assessed in conjunction with other findings and results and may contain passages that require further explanation. We would advise you not to draw your own conclusions from the medical information but to speak to the doctor who is treating you, if necessary.

Ms Jane Sample
Patient number : 12345678

Medical Record

- Pathology results
- Imaging tests
- Lab results
- ECG (electrocardiogram/heart test)
- Ergometry (bicycle test)
- Lung function test
- Quality of life scores
- Operations
- Letters
- Medical glossary

Lab Results

Print

The results of the laboratory tests can be found under Lab Results. The name of the blood component measured is stated in the column 'Measurement'. The column 'Units' shows the value in which the component is expressed. The column 'Reference Value' indicates within which values the value of the component is found in healthy adults in 95% of the cases (source: Farmacotherapeutisch Kompas). The measured values relating to you are stated in the right-hand columns (with a date at the top). An 'L' means that the value measured is lower than the reference value; an 'H' means that the value measured is higher than the reference value. PLEASE NOTE: It is not an immediate problem if a measured value does not fall within the reference values. Your situation is determined on the basis of a combination of results. More information about the various components can be found on the following website: http://www.uwbloedserieus.nl/aanvraagformulier.php (in Dutch).

Measurement	Units	Reference Value	24 Jan. 2014 09:00	2 October 2013 15:50	29 July 2013 09:46	6 May 2013 15:38	13 March 2013 10:15
**Blood transfusion**							
Screening			Neg.	-	-	-	-
**Tumour markers**							
CEA	µg/l	<6	-	2	-	-	-
CA125	kU/l	<35	-	17	-	-	-
**Haematology**							
Haemoglobin	mmol/l	7.5-10.0	7.4 L	7.6	7.1 L	6.6 L	6.6 L
Haematocrit		0.35-0.45	0.37	-	-	-	-
M.C.H.C. (Hb/Ht)	mmol/l	19.0-22.5	20.2	20.1	20.4	19.9	20.8
Leucocytes	10E9/l	4.0-10.5	5.6	7.8	6.2	6.6	4.8
Neutrophil granulocytes	10E9/l	1.8-7.2	2.2	4.2	3.6	3.3	2.0
Thrombocytes	10E9/l	150-400	184	188	196	235	215
**Liver functions**							
Total bilirubin	µmol/l	<16	-	4	6	6	-
Alkaline phosphatase	U/l	<98	-	64	57	71	-

Ms Jane Sample
Patient number : 12345678

Questionnaires

- Fill in questionnaires
- Completed questionnaires

General Quality of Life

Print

We are interested in the state of your health. Please answer all the questions yourself by selecting the answer that applies to you the most. There are no 'correct' or 'incorrect' answers. The information you provide will be treated as strictly confidential. The questions relate to last week (except for questions 1-5, which relate to you how you are feeling right now).

1. Do you have any trouble doing strenuous activities, like carrying a heavy shopping bag or a suitcase?
   - ○ Not at all
   - ○ A little
   - ○ Quite a bit
   - ○ Very much

2. Do you have any trouble taking a long walk?
   - ○ Not at all
   - ○ A little
   - ○ Quite a bit
   - ○ Very much

3. Do you have any trouble taking a short walk outside of the house?
   - ○ Not at all
   - ○ A little
   - ○ Quite a bit
   - ○ Very much

- Lab results
- ECG (electrocardiogram/heart test)
- Ergometry (bicycle test)
- Lung function test
- Quality of life scores
- Operations
- Letters
- Medical glossary

If you have not answered all the questions, some parameters will not show a score. The meaning of a number of subjects under the column 'Parameters' are now explained in detail below.

- General QOL means general quality of life. This score indicates how you are feeling in general. It concerns all the physical, mental and social aspects combined and how you experience them.
- Physical means physical functioning (walking, lifting things, etc.).
- Role means role functioning (work, daily activities).
- Cognitive means cognitive functioning (concentration and memory).
- Emotional means emotional functioning (anxiety, irritability, etc.).
- Social means social functioning (social activities and family life).

Information about the various subjects (parameters) plus tips can be found under Information - 'Complaints and Symptoms'.

General Quality of Life

Although the doctors who are treating you can also view these scores, we would advise you to always talk about your complaints and/or concerns during your appointments at the AVL.

Parameter	Unit	27 March 2014 11:45:53	27 March 2014 12:13:00
General QOL	0-100; (100 = best)	100	0
Physical	0-100; (100 = best)	100	0
Role	0-100; (100 = best)	100	0
Emotional	0-100; (100 = best)	100	0
Cognitive	0-100; (100 = best)	100	0
Social	0-100; (100 = best)	100	0
Fatigue	0-100; (0 = best)	0	100
Sickness/vomiting	0-100; (0 = best)	0	100
Pain	0-100; (0 = best)	0	100
Shortness of breath	0-100; (0 = best)	0	100
Sleep	0-100; (0 = best)	0	100
Appetite	0-100; (0 = best)	0	100
Constipation	0-100; (0 = best)	0	100
Diarrhoea	0-100; (0 = best)	0	100
Financial impact	0-100; (0 = best)	0	100

First | Previous | ~~Next~~ | Last

Ms Jane Sample
Patient number : 12345678

Patient education

Fatigue

Print

Many people who have, or have had cancer suffer from fatigue. This type of fatigue feels different to simply being tired and can occur suddenly. Fatigue could be a side effect of treatment(s) but you may also continue to suffer from fatigue on completion of the treatment(s). Although fatigue could be caused by the treatment, other factors could cause the fatigue complaints to continue.

Continued fatigue could also be caused by a deteriorated condition, a disrupted sleeping pattern and a disrupted day structure. However, cancer is an illness that may be emotionally draining. This not only includes fretting, uncertainty, restlessness, anxiety and distress but dealing with everything you have gone through since being diagnosed with cancer. These emotions consume energy and can cause you to sleep poorly, in turn, causing fatigue. If you are tired, those around you may sometimes respond in a 'patronising' manner. People will take tasks off your hands, and thus encourage inactivity. Always remember that it will benefit you to start doing things again yourself.
When carrying out your daily activities fatigue can be rather annoying. It might restrict you in your work, you may not have any energy to go to a birthday party or do the washing up. There are various things you can do to reduce fatigue.

Advice

- Ensure rest periods and regularity in your life. This will help you avoid taking on too many activities (overdoing it) at times when you are feeling well. If you overexert yourself at times when you are feeling well, it will often take you several days to recover afterwards. You will thus continue to be fatigued.
- Only undertake activities that give you energy.
- Ensure you maintain a proper balance between resting and exercise.
- Try to exercise at home, or if you are able to, go for a walk or a bicycle ride.

Ms Jane Sample
Patient number : 12345678

Questionnaires

- Fill in questionnaires
- Completed questionnaires

Recommended exercise

Print

We are interested in the type(s) of physical activity people perform in their daily lives. The questions relate to your physical activities in the past seven days. Please answer all the questions, even if you believe that you are not physically active.
Think of activities you carry out at work, at and around your home, activities taking you from one place to another and recreational, training and sports activities in your leisure time.

Please include all the activities you performed in the past seven days and only the activities that you carried out for at least ten minutes.

Heavy physical activities are activities requiring a great deal of physical effort and make you breathe faster (such as lifting heavy loads, digging the garden, aerobics or cycle racing. Moderately intensive physical activity makes you breathe slightly faster than usual (such as carrying light loads, cycling at a normal speed or playing doubles tennis). Do not include walking in this section.

On how many of the past seven days have you performed heavy physical activities?

Next | Cancel

Ms Jane Sample
Patient number : 12345678

Keep fit

- Keep fit

Keep fit

Print

Because exercising can have numerous positive effects, we believe that it is essential to highlight this topic here. This section provides information and personal advice on exercising based on the questionnaire you have completed on your exercise behaviour.
The recommendations are tailored to each patient's individual situation as far as possible but should not be considered as a substitute for a consultation or treatment by a care provider. If you are suffering from specific complaints or physical discomfort, do not simply follow the recommendations, but please contact your doctor.

Recommendations

Dutch Norm for Healthy Activity
You currently meet the exercise standard of a minimum of 150 minutes of moderately intensive exercise per week. This means you will benefit from its health promoting effects. Excellent!

CONTINUE TO ASK FOR SUPPORT
Even if you have been physically active for some time, it is still essential to talk about this with your family or friends. You can also ask them for help if you have not been able to exercise for a while. Other people can sometimes even spur you on to keep exercising.

VARY YOUR PHYSICAL ACTIVITIES
Vary your physical activities to ensure they remain attractive and you do not get bored with them. Your activities can be varied in several ways. You could opt for another type of activity rather than your usual activity, or a different walking or cycling route.

Keep fit

The table below shows your physical activity (the amount of activity). The information is based on your own answers (to the questions answered on MyAVL).

The column ‘parameter’ shows the type of exercise, i.e. walking, moderate activity (carrying on walking a long distance, for instance), heavy activity (such as running) and moderate to heavy activities. The latter refers to the total of moderate to heavy activity combined. The value of ‘moderate to heavy’ also shows whether you meet the Dutch Standard for Healthy Exercise of a minimum of 150 minutes of moderately intensive exercise per week.

The column ‘unit’ shows the amount of exercise in minutes per week. The other columns show your actual physical activity on the date on which you completed the questionnaire. If you have not answered any questions about physical activity, no information will be shown in or added to the table. This applies to the following cases:

1. You have indicated that you are due to undergo, or have recently undergone an operation.
2. You have indicated that you are taking part in a supervised exercise programme.
3. You have indicated that special circumstances apply to your health in relation to exercising.

**Parameter**	**Unit**	6 January 2014 10:24:28	6 January 2014 13:20:22	19 June 2014 12:05:34
Walking	Minutes/week	0	0	100
Moderate activity	Minutes/week	0	0	150
Heavy activity	Minutes/week	0	0	120
Moderate and heavy	Minutes/week	0	0	270

First | Previous | ~~Next~~ | Last
